# Bio-Mimetics of Disaster Anticipation—Learning Experience and Key-Challenges

**DOI:** 10.3390/ani3010274

**Published:** 2013-03-19

**Authors:** Helmut Tributsch

**Affiliations:** Bio-Mimetics Program, Carinthian University of Applied Sciences, Europastrasse 4, 9524 Villach, Austria; E-Mail: helmut.tributsch@alice.it

**Keywords:** earthquake prediction, disaster anticipation, ancient wisdom, animal anomalies, Tangshan earthquake, temperature anomalies

## Abstract

**Simple Summary:**

Starting from 1700 B.C. in the old world and up to recent times in China there is evidence of earthquake prediction based on unusual metrological phenomena and animal behavior. The review tries to explore the credibility and to pin down the nature of geophysical phenomena involved. It appears that the concept of ancient Greek philosophers in that a dry gas, pneuma is correlated with earthquakes, is relevant. It is not the cause of earthquakes, as originally thought, but may be an accompanying phenomenon and occasional precursor. This would explain unusual animal behavior as well as thermal anomalies detected from satellites.

**Abstract:**

Anomalies in animal behavior and meteorological phenomena before major earthquakes have been reported throughout history. Bio-mimetics or bionics aims at learning disaster anticipation from animals. Since modern science is reluctant to address this problem an effort has been made to track down the knowledge available to ancient natural philosophers. Starting with an archaeologically documented human sacrifice around 1700 B.C. during the Minoan civilization immediately before a large earthquake, which killed the participants, earthquake prediction knowledge throughout antiquity is evaluated. Major practical experience with this phenomenon has been gained from a Chinese earthquake prediction initiative nearly half a century ago. Some quakes, like that of Haicheng, were recognized in advance. However, the destructive Tangshan earthquake was not predicted, which was interpreted as an inherent failure of prediction based on animal phenomena. This is contradicted on the basis of reliable Chinese documentation provided by the responsible earthquake study commission. The Tangshan earthquake was preceded by more than 2,000 reported animal anomalies, some of which were of very dramatic nature. They are discussed here. Any physical phenomenon, which may cause animal unrest, must involve energy turnover before the main earthquake event. The final product, however, of any energy turnover is heat. Satellite based infrared measurements have indeed identified significant thermal anomalies before major earthquakes. One of these cases, occurring during the 2001 Bhuj earthquake in Gujarat, India, is analyzed together with parallel animal anomalies observed in the Gir national park. It is suggested that the time window is identical and that both phenomena have the same geophysical origin. It therefore remains to be demonstrated that energy can be released locally before major earthquake events. It is shown that by considering appropriate geophysical feedback processes, this is possible for large scale energy conversion phenomena within highly non-linear geophysical mechanisms. With satellite monitored infrared anomalies indicating possible epicenters and local animal and environmental observations immediately initiated, the learning experience towards an understanding of the phenomena involved could be accelerated.

## 1. Introduction

Predicting earthquakes in any reliable way with the intention to save people and to safeguard potentially dangerous industrial installations is a significant challenge for the future. Even though traditional earthquake precursors from popular tradition have been consistently reported since antiquity and from all major civilizations, and even though more and more compilations and evaluations appear in the literature [1,2,3,4,5,6,7,8,9,10,11,12,13], the possibility of considering atmospheric or animal precursors is still discussed in a very controversial way in the geophysical community. If traditional earthquake precursors from the popular tradition could be accepted as reasonably relevant, modern experimental tools could help us to design appropriate experiments to identify responsible physical-chemical mechanisms with modern equipment. It is for this reason that additional evidence on possible precursors and their practical use for earthquake prediction would be highly helpful. An interesting study case has been identified by us in connection with a severe earthquake which hit the Greek island of Crete around the year 1700 B.C. 

## 2. A Human Sacrifice just Preceding a Severe Earthquake

At that time, around 1700 B.C., the strong earthquake concerned destroyed the capital Knossos of the Minoan civilisation with its palace, which became known worldwide for its Minotaur and its labyrinth. In fact all stone buildings from that time, on the island of Crete, were essentially destroyed. The palace of Knossos was rebuilt later, however a sanctuary which was situated seven kilometres further south on a hillside below the mountain Jouchtas, the legendary grave of the Cretean god Zeus, in a location named Anemospilia, was not rebuilt. The sanctuary completely collapsed and stayed untouched for 3,700 years. Then the attention of Greek archaeologists was drawn to this place. From there one has a magnificent view over the central territory of the Minoan civilisation. Everything in the small sanctuary, which consisted of three parallel rooms, oriented towards the original capital Knossos, and an entrance room, extended in front, has been conserved, as it was buried by falling wood beams and walls. The central room was reserved for the god-statue and sacrificial donations. Also the room to the right was full of donations, mostly fruit and grain. In the room to the left there was a stone platform, a table, for the preparation of the sacrifice. As well conserved paintings from the Minoan culture clearly show, on such a table during a sacrifice the artery near the throat of a chained bull was cut in order to collect the blood in a sacrificial vessel, especially intended for this purpose, which was then carried to the statue of the god. Four slain humans, the conserved remains of a large statue of a god in the central room, and around 400 pieces of pottery were discovered on the excavation site. Under the careful strokes of brushes by archaeologists and the critical eyes of criminologists a dramatic scene was brought back to life, which documented the intentions of the people concerned, immediately before the terrible earthquake. All this was later documented in a very detailed account published in the Magazine of the National Geographic Society [14].

The temple servant, who was supposed to carry the sacrificial chalice with the blood to the central room of the sanctuary was slain on the way there and the blood-chalice was crushed into 105 pieces (which were carefully reassembled by the archaeologists).

In the side room, close to the stone-made preparation table, the slain high priest was found with an exceptionally rich decorated long sacrificial knife. Beside him a priestess was smashed to the ground. The surprise was that, on the stone-table no chained bull was found, but a confined human, an approximately 18 year old young man. The immobility of his limbs was deducible from his position. His body was, when the ceiling of the sanctuary hit him, in part already free of blood. This was clearly determined by the criminologists on the basis of the colour of the bones. 

In the last few minutes before this severe earthquake struck, the high priest apparently tried to stop the impending calamity by a human sacrifice. Or was the coincidence with the earthquake just chance?

For the Minoan culture a human sacrifice was a possible, but not at all a common ritual. The story of the Minotaur, a creature half bull, half man, living in the Labyrinth, is an important example from the far past. The city of Athens had every year to send seven youngsters and seven girls for sacrifice, until the hero Theseus, supported by the daughter of the King Minos, Ariadne with her wool clue, killed the Minotaur. In Knossos another example of human sacrifice was found. In a building dating back to 1500 B.C., which had been destroyed by fire, ritual vessels were found, which suggested that it was a sanctuary. Here the bones of two children were found with cuts, which suggested that they were slaughtered like the bulls and that the flesh had then been taken from their bones.

Though the ritual may be rare and reserved for very special occasions, the earthquake in 1700 B.C. has obviously immobilized the ritual of a human sacrifice.

What can be learned from such a coincidence with the very strong, 3,700 year old earthquake?

First it would be entirely unrealistic to believe, that the very rare case of a human sacrifice would have had another purpose rather than the impending earthquake. The chance of such a coincidence would have been far too small. Furthermore, it happened not in a town centre but in a remote hill sanctuary, which was obviously designed to deal with natural phenomena. The population of the seven km distant town of Knossos was apparently warned, because practically no victims of this severe earthquake were found in ruins elsewhere. Or were they removed later?

Assuming, that there was a relation between sacrifice and earthquake, the high priest must have observed something, which convinced him that a natural disaster of this type was imminent. Smaller foreshocks could not have been the reason. In the Aegean Sea there are far too many small earthquakes, without a large one following, to justify a human sacrifice. There must have been another phenomenon, which convinced the priest of a large impending earthquake. It must have been apparent hours before, because it is practically impossible to prepare a human sacrifice within a few minutes. Already the time period needed to take such a serious decision must have been quite long. Then comes the problem of finding a victim and transporting it to the temple. Who was the 18 year old male victim? Was it a slave or was it a voluntary victim from the temple community, who was chained to the sacrificial platform? Something must have escalated in nature to justify such a desperate step as a last resort.

This archaeological evidence of natural disaster anticipation does not stand alone in Greek antiquity. In 1650 B.C. the volcano of Santorin erupted and destroyed this and neighbouring islands. When excavating the ash- and lava-buried Minoan settlements archaeologists expected human remains similar to those found in Pompeii. The fact is that no human victims were found, even though approximately 30,000 people are believed to have lived on Santorin island. Even the amphorae used for food storage were found to be empty. Clearly people had all left safely with their essential belongings before the disaster. 

There is written evidence for earthquake anticipation during antiquity: The writer Cicero reports on earthquake prediction, which occurred in the 6th century B.C. in Sparta. One day the natural philosopher Anaximander told the people of Lakädemonia that an earthquake would arrive and asked them to leave their houses and the town and to camp during the night outside in full armament. His recommendation was followed, the entire town collapsed and a large piece of rock came down from the mountain of Taygetos [15]. The natural philosopher had apparently drawn relevant information from the environment, which appeared at least one day ahead of the earthquake. He must have been sure of the precursors, otherwise he would have risked making himself look ridiculous. 

In fact, earthquake precursors were known and described during antiquity. The Roman natural scientist Pliny the Elder cites four earthquake precursors: foreshocks, turbid water, exited animals, a strange fog [16]. The first two are known to modern geophysical science, the latter two are not yet recognized and will be subject of this study. 

## 3. Religious Earthquake Related Practices

Since the Minoan sanctuary, which was destroyed by the earthquake 1700 B.C., was situated on the northern slope of a mountain, Jouchtas, associated with the most powerful god Zeus (later named Jupiter by the Romans), the sacrificial ceremonies performed in this sanctuary should have been related to worship associated with Zeus or one of his descendents. According to Greek mythology Zeus had god like or semi-god like children with approximately 37 women. Among them, there is only one god, who is immediately interesting for our investigation, since he alone is responsible for the powers of nature and of nature’s fertility. It is god Dionysos or, as the Romans later knew him, god Bacchus. Mythology, according to the most prominent tradition, tells that Zeus (Jupiter) had his son Dionysos (Bacchus) with Semele, the daughter of the Theban King Kadmos. He was educated by the nymphs of Nysa and later by the strange, bald fellow Silen. Now it becomes interesting: when grown up Dionysos (Bacchus) married the daughter Ariadne of King Minos from Knossos in Crete. Coincidentally, Ariadne was the woman who helped Theseus to defeat the terrible Minotaur by providing the wool clue and a sacred sword. This means, that the god Dionysos (Bacchus) is actually linked with Knossos. It may therefore indeed be that the Dionysos/Bacchus cult, for which we have later written and pictured records, is actually a late continuation of the discussed ancient ceremonial practice involving a human sacrifice in 1700 B.C. 

A first strong support comes from Roman sources, indeed associating the Bacchus cult with ritual human sacrifices. Because of these human sacrifices and because of ecstatic ceremonies and orgies during special festivities (bacchanalia), the senate of Rome in 186 B.C. severely restricted these activities (senatus consultum de bacchanalibus). Later the Bacchus cult became therefore mostly associated with wine consuming excesses and the pleasures of life. Such an attribute gave it a superficial and easy-going appearance. This may not always have been the case. Ceremonies leading to ecstasy are known from many natural tribes and are typically associated with now quite well understood shamanic practices. It has always been a practice aimed at assuming contact with the other world, often in an effort to recognise impending danger and to control the future. 

## 4. Bacchus and the Power of Natural Forces

**Figure 1:**
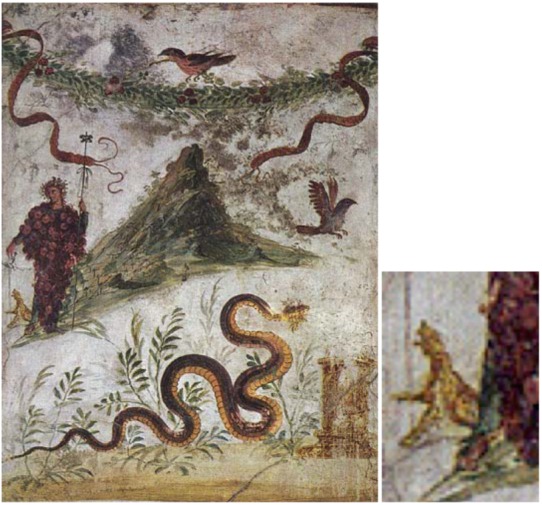
Bacchus and the volcano Vesuvius. Archaeological National Museum Naples, Italy. From Pompeii, House of the Centennial. The traditionally given interpretation of Bacchus together with animals representing the fertility of the land is challenged. It is proposed that excited animals are seen in the company of Bacchus, anticipating a natural (earthquake) disaster. The right hand figure is a magnification of the howling dog behind Bacchus.

There are many figurative representations of Bacchus associated with an easy-going way of life. I found only one showing him as the lord of natural forces (Figure 1). It is a painting of 140 × 101 cm found in the volcanic ruins of Pompeii. It shows Bacchus, dressed with a large wine grape together with animals in front of the volcano Vesuvius. With its animals and plants the painting is supposed to express his responsibility for the fertility of the country. 

**Figure 2:**
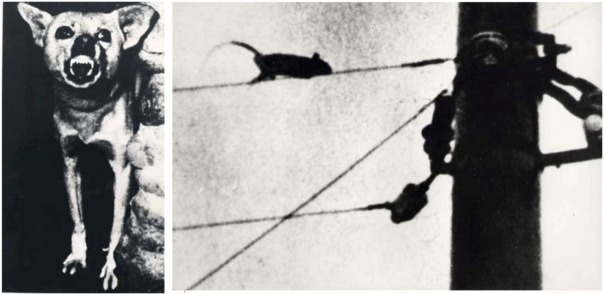
Pictures of an excited dog, and of a fleeing rat, which were taken prior to the M = 7.2 Sungfan-Pingwu earthquake from 1976.

However, remembering that Bacchus is the traditional Lord of natural forces, and since a volcano reflects a real earthquake danger, we may look at this scene and especially at the animals in an entirely different way. Pliny the Elder, who coincidentally was killed during the same volcanic eruption which eradicated Pompeii, and which conserved this beautiful painting (which Pliny the Elder may have known), reported, as already mentioned, four phenomena, which could precede an earthquake [16]. One was that “even birds don’t remain sitting quietly”. The painting from Pompeii reproduced in Figure 1 shows two of these restless birds. Besides it shows an agitated snake in full movement. I have named my book written long ago on earthquake precursors from the popular tradition “When the snakes awake” [6], because so many reports from all over the world deal with snakes escaping from the underground, prior to earthquakes. The most conspicuous detail, however is a howling or at least a very aggressive dog just to the left behind Bacchus, amplified to the right (Figure 1). An aggressive dog does not fit at all into a painting, supposed to express the fertility of a natural environment. It is, however, a perfect symbol for a threatening earthquake or volcanic eruption. Very aggressive and frightened dogs have been consistently reported as precursor phenomena preceding major earthquakes. In relation to the aftershocks of the Messina earthquake of 1783, for example, dogs were barking so intensively that the authorities ordered them to be shot. The stressed people could not tolerate the noise any longer. During the Chinese earthquake initiative of the sixties and seventies of the last century, based on popular earthquake precursors, photographs of anomalous animal behaviour before earthquakes were taken. Among these a picture of a dog was also taken prior to the M = 7.2 Sungfan-Pingwu earthquake from 1976. It is reproduced in Figure 2 and matches the behaviour of the dog behind Bacchus in Figure 1.

The painting of Bacchus and Vesuvius (Figure 1) should therefore be reinterpreted: It shows Bacchus, the Lord of natural forces, in relation to earthquakes and volcanic eruptions. His arrival, as lord of natural forces, and thus the arrival of a natural disaster, is announced by an anomalous behaviour of animals, such as that of snakes, birds and dogs. The painting had probably been produced while memorizing the events during another quite severe earthquake, in 62 A.D., 17 years before the final destruction of Pompei, which occurred in 79 A.D. A marble bas relief from the house of L. Caecilius Jucundus also shows the events during this earlier earthquake together with two frightened, fleeing donkeys.

For our initiative with respect to ancient earthquake knowledge the result is that one strategy of earthquake anticipation known within the Bacchus cult was apparently the observation of anomalous animal behaviour, as depicted in Figure 1. This is entirely consistent with complementary information. In ancient Rome and also within the Etruscan tradition (which is known to have been significantly influenced by the Greek tradition) this animal phenomenon was taken seriously. In the Roman literature many cases of unusual or untimely animal behaviour, such as the unexpected appearance of snakes, of “danger talking” or simply “talking” animals, of nocturnal birds during the day, or of wild animals, such as wolves, in urban areas, were reported as indication of special danger [6] When “talking” animals, probably animals excessively communicating in their own voice, were reported, the Roman Senate had to conduct its sessions in the open. The role of geese on the Roman Capitol as danger forecasting animals is also a well known example.

Another interesting fact is that the Dionysos/Bacchus cult has always been associated with processions and theatre-like presentations in honour of the god (which in Greece finally resulted in the classical Greek theatre). Processions also played a non-negligible role in connection with earthquake danger. When wolves penetrated to the Roman Capitol in the year 458 B.C. an expiation ceremony was ordered. People had to leave their houses and to join a procession with decorated cattle. By the way, such a procession is also depicted on the above mentioned marble bas relief from Pompei (besides the shying donkeys, which may indicate, that the procession with decorated bulls had already started earlier). The town had to be circumvented three times by the procession and at the end of this time consuming ceremony, the animals were sacrificed to the god. If an earthquake were to have hit, most people would have been in the open air and would have survived. Earthquake prediction attempts were hidden in religious practices, against which nobody could argue, and if the earthquake did not come, it was god`s will or the result of a successful ceremony aimed at avoiding it. 

When extrapolating back to the initially described human sacrifice preceding a large earthquake in 1700 B.C., we may consequently reach the following impression of what happened during the earthquake tragedy in ancient Crete: The priests serving the god responsible for natural disasters recognized atmospheric and animal anomalies and decided to act. While the numerous population of the capital was engaged in a longer lasting procession in the open air, the chief priest and his aids were on duty in the temple near the statue of god, performing a human sacrifice to fend off the impending earthquake. They were killed by the natural disaster. 

## 5. What Earthquake Precursors did Ancient Greek Philosophers Know?

The second strategy, which should be followed to find an explanation for the anticipation of an earthquake in 1700 B.C. is to follow and analyse recorded ancient knowledge on earthquakes. Starting from the 7th century B.C., over a dozen antique philosophers or scientists theorized about earthquakes: among them Thales of Milet (appr. 650–560 A.D.), Anaximenes (second half of 6th century B.C.), Anaxagoras (appr. 500–428 B.C.), Demokritos (born around 460 B.C.), Antiphon (490–411 B.C.), Aristotle (384–322 B.C.), Theophrastos (371–287 B.C.), Straton of Lampsakos (320–270 B.C.), Poseidonios (135–51 B.C.), Pliny the Elder(24–79 A.D.), Seneca (55 B.C.–40 A.D.), Ammianus Marcellinus (second half of 4th century A.D.). They tried to find natural reasons and forces for the occurrence of earthquakes, even though religion suggested angry and punishing gods as the origin of such disasters. There was no contradiction: natural forces were also considered to be controlled by a god such as Dionysos/Bacchus. 

It is reasonable to assume that these early natural philosophers based their theories and their knowledge on earthquake precursors, on then commonly discussed and popularly recognized real observations of phenomena associated with earthquakes. Due to the low population density and the simple way of life, the environment during antiquity was still largely unpolluted, so that meteorological and biological earthquake precursors could better be distinguished. People also had more time to observe nature.

Different explanations have been advanced during antiquity to explain why the earth is shaking. However the most important, widely distributed and persistent idea was that gases, called “pneuma” were involved in the build up and generation of earthquakes. They may have entered the earth from outside, or may have formed inside. There must have been practical evidence for these gases. Something must have been observed that suggested gases were escaping before and during earthquakes.

Anaxagoras (according to Aristotle [17]), in the 5th century B.C. already assumed that a gaseous ether forces its way through the porous earth and through the contained air from below the earth’s disc thus generating earthquakes. Aristotle based his earthquake concept on this pneumatic mechanism communicated by Anaxagoras. His elaborated ideas have been conserved in his famous book on meteorology [18]. Basically he assumes, that a compressed dry and warm gas, pneuma, escapes from the earth thus producing earthquakes. The same origin has also a sign that according to Aristotle used to precede the earthquake: “In clear weather during the day and shortly after sunset, a narrow long stretched cloud becomes visible, like a line drawn by a ruler, because the pneuma is disappearing. What remains is a kind of surf line of the air-sea”. Aristotle also describes some of the fog’s properties: “Besides the weakening of the sun and the (relative) darkness that comes about without clouds, the calmness and great cold that occasionally occur before earthquakes, that happen in the morning, confirm the cause”.

The strange cloud or fog, which appears before the earthquake, and is described by Aristotle, has also received attention during a later historical event and Pliny the Elder explicitly mentions it as one of four possible earthquake precursors [16]. The Roman writers Livius [19] and Cicero both report of the strange fog, which occurred in 217 B.C. at the Trasimenian lake, just as the Romans and Carthaginians were involved in a fierce battle. Their information goes back to L. Coelius Antipater, who was living at the time of this battle. The report says that the fog was much more dense on the plain than in the mountains. The Roman army, commanded by C. Flavinius, proceeded within the fog towards the camp of their enemies while on the other hand Carthago’s mobile forces on the hills, could see each other and coordinate their attack. While the battle was fought a severe earthquake hit, which also destroyed numerous settlements in ancient Italy. However, it was reported that the warriors engaged in fighting barely noticed it. According to this description the strange fog was already present before the earthquake and was a reason for the trapping and the defeat of the Romans.

Remarkably, and typical of all antique understanding of earthquakes, Theophrastus, (as explained in [20,21]) discusses this phenomenon in his Meteorology after dealing with clouds, rain, snow and wind. Earthquakes were considered to be deeply linked to atmospheric phenomena.

The pneuma theory of earthquakes received further studies from Straton of Lampsakos, the teacher of Ptolemaios II, from Poseidonios, (as reported by Seneca) and from Ammianus Marcellinus, in his “Res gestae” [22,23].

Also during all the Middle Ages the “pneuma” earthquake theory of escaping gases continued to be respected as a reasonable explanation, for example by Albertus Magnus or Thomas Aquino. We are thus dealing with an ancient scientific concept on earthquake phenomena that has amazingly survived at least during two millennia. 

## 6. Ancient “Pneuma”—Theory Based on Really Observed Earthquake Precursors?

With the development of modern science and the establishment of the geo-tectonic concepts of earthquake events, the “pneuma”-concept of gases escaping from the ground prior to and during earthquakes became an imaginative invention of scientifically naive early natural philosophers. The now accepted and verified notion that escaping gases are not responsible for earthquakes however, obscured the possibility that they still could participate in the earthquake event as precursors or accompanying phenomena. For this reason nobody really cared to look with modern scientific tools for escaping “pneuma”-gases prior to or during earthquakes. The age-old experience and knowledge of early scientists in relation to a possible side phenomenon of earthquakes was simply ignored. The aim of this paper is to show that it may also be relevant for understanding unusual animal behaviour before earthquakes. 

What ancient natural scientists really could have experienced during their lifetime or could have communicated as ancient knowledge, or what the high priest from the discussed earthquake disaster on Crete in 1700 B.C. could have observed, may best be illustrated with a report from a more recent earthquake event on another Mediterranean island: During a visit of participants of the 3rd International Conference on Earthquake Precursors (ICEP), Naples (Italy, September 1988), to the site of the town Gibellina in Sicily, which had been completely destroyed by an earthquake in January 1968, the author asked eyewitnesses whether precursors had been observed and he got surprising information from a quite reliable source [24]. The quake, which killed 5,000 people in the town, happened in the early hours of the day (3 A.M.). It was preceded by some minor shocks, which occurred the afternoon before. With the first of these shocks, snow started to fall and it became very cold. This is a very unusual weather condition for Sicily where citrus fruits are usually harvested during that period. In addition, a strange fog started to appear, the distribution of which became most spectacular at sunset. While the sky remained grey, a thin bright red strip became visible above and all along the horizon. The intensive red colour was apparently the well known consequence of the scattering of blue light at scattering centres, as frequently seen during sunset. The rim of the cloud was not sharp but appeared to be diffuse. Such a pronounced phenomenon had not been seen before in that locality. People also reported to have felt weak and depressed. At that time an unexplainable nervousness in geese had already been observed. Due to the severe cold, people did not stay in the open after sensing the modest foreshocks and were hit by the severe quake during the night. According to witnesses, approximately 20 minutes before the disaster dogs started to howl and to roam around. Also, donkeys were observed to break out from their pens. Atmospheric and animal anomalies were parallel phenomena. They apparently had the same origin.

If we tentatively take these singular observations of pre-earthquake phenomena associated with the Gibbelina-quake for granted and compare them with what the ancient naturalists were reporting, we immediately realize an astonishing similarity. A metrological situation arises, which resembles a smog laden heat island around a densely populated modern city. There is the strange fog, which may dim the sun light. And there is the long stretched thin cloud on the horizon, which, according to Aristotle, “is visible during the day or shortly after sunset”. Aristotle also compares it with a surf-line. This suggests that it is seen close to the horizon, like the haze produced by smog. The narrow smog pattern above the horizon, which assumes a reddish appearance during sunset is nowadays a well known phenomenon seen around large urban centres. The “pneuma” gas which was seen escaping from the earth was considered to be warm and dry. Such a condition has frequently been reported as typical “earthquake weather” from many earthquake regions of the world [6]. Sensitive people feel uneasy. A similar situation is felt in smog ridden cities. The high aerosol concentration and its higher viscosity may also favour the still in the air. Alternatively the unusual cold, which precedes earthquakes in the early morning (when no greenhouse warming may be expected), was also emphasized by Aristotle. The explanation is that the air becomes better at heat conducting by carrying aerosols and cold feels colder (in absence of heating sunshine) because the warm body looses heat faster. 

The appearance of gases and the accompanying alteration of the earth near atmosphere together with an approaching earthquake receive strong support from popular tradition. An analysis of 78 reported documents on relevant earthquake precursors from different civilizations [6] allowed a distinction into three classes of phenomena. They are atmospheric anomalies like fog where it is typically not observed, unusual animal behaviour and luminous phenomena. All three could be explained with the hypothesis that (charged) aerosols may appear in the air above ground before and during an earthquake. Apparently, before some large earthquakes the earth starts degassing already during the preparative stage of the earthquake. 

Such a phenomenon would be imaginable as a consequence of pressure changes underground. It could induce a change of chemical equilibrium, of pressure induced electrical phenomena [6], or of other pressure induced charge separation processes [25]. However, is it gas and aerosol emission prior to earthquakes which is affecting animal behaviour? Today such phenomena are not yet considered as geo-physically accepted earthquake precursors (e.g., [26]).

Animals, depending on the species, are known to respond to many physical and chemical signals in a much more sensitive way than humans. They can be sensitive to very different signals. They may respond to different signals before earthquakes. Most comparative information on animal behaviour before earthquakes was compiled during the Chinese initiative for earthquake prediction on the basis of animal and environmental precursors, which occurred during the sixties and seventies of the past century.

## 7. Chinese Experience with Animals Prior to Earthquakes

**Figure 3:**
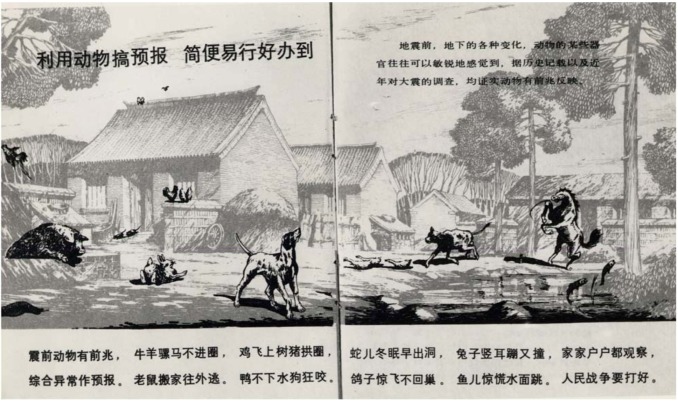
Chinese flyer, distributed to country people with the intention of explaining the behaviour of animals prior to earthquakes, which should be observed.

**Figure 4:**
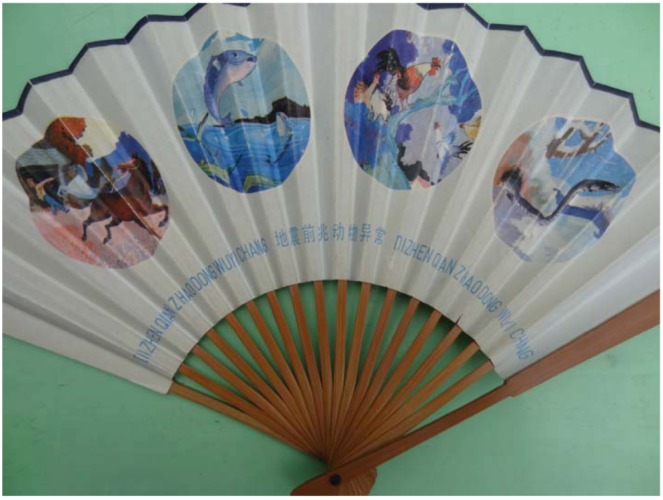
Fan, depicting animal behaviour before earthquakes, with information on how to react (backside), distributed to country people in China in support of a collective earthquake watch.

The Chinese initiative for earthquake prediction, which took place fifty years ago following a political decision, was based on a broad cooperation of peasants and agricultural and farm workers, who were informed and trained with illustrated leaflets and fans on how to judge unusual animal behaviour before earthquakes (Figure 3, Figure 4). Unusual appearances of rats, insects, birds and snakes belonged to such phenomena, as well as frightened horses and cattle or pigs breaking out from their pens. More than 50 different animals were cited to show unusual behaviour before earthquakes and a clear graduation in the degree of sensitivity was observed. This wide spread sensitivity of animals to earthquakes is surprising, since animals have very differently evolved senses for environmental signals. Cave dwelling animals, such as bats, weasels or snakes were found to be the most sensitive ones. Also rats belong to this group. Then follow fish and frogs. The next group comprises domestic animals such as dogs, cats, poultry, cattle and horses. 

It has been argued [6] that earthquakes are not frequent enough to explain evolution of an earthquake sense in animals. Why should insects and birds also be frightened by earthquakes? Nevertheless, we have discovered a surprising parallelism to animal behaviour before strong storms [6]. Animals may sense signals, which suggest the arrival of a severe weather condition. As a consequence, cave and burrow dwelling animals escape into the open air in an attempt to avoid supposed flooding of their habitat. Insects may flee from large rain drops and hail. Domestic animals may try to avoid their pens due to the presence of an oppressive air or because they prefer to feed before the expected arrival of severe weather. However many questions remain unanswered. Chinese scientists, who participated in the earthquake prediction program admitted that they do not have reliable explanations for the abnormal animal behaviour before earthquakes. Their plan simply was to gather information on an empirical basis over a long time period. 

The Chinese authorities, thus proceeding in an entirely empirical way, used to give an alarm, when the incoming reports on animal and environmental anomalies escalated. They have claimed to have predicted up to 10 earthquakes, the best known being the earthquake of Haicheng on 4 February 1976. It was a magnitude 7.3 earthquake, shortly ahead of which the population was evacuated from their houses into the snowy open landscape. Characteristic longer term indications for the arrival of this earthquake were snakes which left the underground to freeze on the icy surface. I myself have seen many of these amateur photographs, when I visited the Institute of Biophysics in Beijing in 1988. Abnormal animal behaviour in the Haicheng area was observed in 800 cases and concerned 30 animal species (cited in [27]). 

Another large destructive earthquake, five months later, on 27 July 1976, the magnitude 7.8 Tangshan earthquake, however has not publicly been anticipated. It occurred at 3.42 A.M. and caused at least 240,000 deaths, according to other Chinese sources 655,000. Mainly because the prediction did not work and due to the internationalization of Chinese earthquake research the interest in systematically studying abnormal animal behaviour for earthquake prediction had ceased. In fact, the failure to predict the Tangshan earthquake was taken as demonstration of the incapacity of a prediction strategy based on mass participation in recognizing animal and other traditional precursor phenomena (e.g., [28]). The situation among professionals may also be characterized by the following opinion [29]: “The value of mass participation was called into doubt when China failed to predict the Tangshan earthquake. The mass participation national strategy declined in the 1980s as predicting earthquakes became the task of professionals who strongly believe that earthquakes cannot be predicted”.

## 8. The Tangshan Earthquake was Preceded by Significant Animal Anomalies

According to the information obtained by the author in 1988 in the Institute of Biophysics, Chinese Academy of Science, in Beijing the failure of Tangshan earthquake prediction was to a large extent caused by political turbulences during July 1976 between the left wing “gang of four” and the pragmatic wing of the communist party around the termination of the cultural revolution. Personalities responsible for giving earthquake alarm were in part simply removed from their positions and could not act. Other opinions, however, also exist and there are detailed reports on the earthquake experience in China which could be considered as a reference (e.g., [30,31]). 

**Figure 5:**
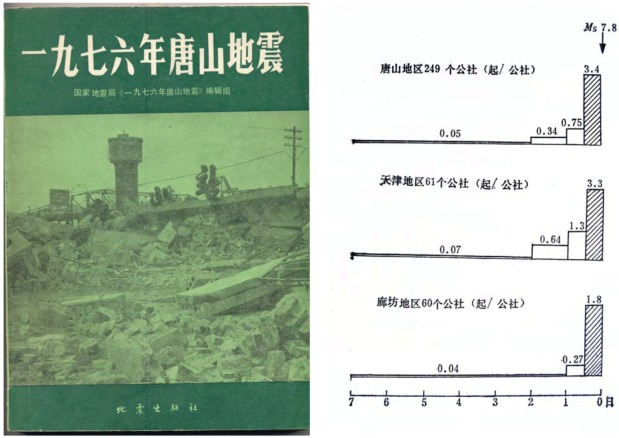
The Chinese report on “The Tangshan Earthquake of 1976” [27] with documentation of the observed unusual animal behaviour. The right graph shows the increase of unusual animal behaviour towards the earthquake event (M = 7.8). The horizontal scale indicates days before the earthquake. The chinese writing on the right picture means: top: District of Tangshan (249 people’s comunes). Center: District of Tianjin (61 people’s comunes). Below: District Langfang (60 people’s comunes).

Two days after the disastrous Tangshan quake researchers from 16 Chinese research institutions formed a research group and started studying many aspects of the earthquake including animal precursors. They published a 459 page thick report on “The Tangshan earthquake of 1976” during 1982 [27]. Chapter XII of this compilation was dedicated to abnormal animal behaviour. Since this publication is quite unknown in the western world, and because it is politically and psychologically very relevant for the discussion of animal precursors, its results are shortly discussed here (Figure 5).

On the basis of well defined standards and geographical patterns reported animal phenomena from 412 people’s communes spread over 60 districts were studied during this initiative of the Chinese earthquake study commission. The working force consisted of members of the biological and chemical seminary of Nankai University, the Pedagogical University of Tianjin and of the Geological Research Institute of Yejin. Most cases of abnormal animal behaviour concerned 30 animal species with abundant presence in the region. As many as 2,202 cases of abnormal animal behaviour were identified in the region affected by the earthquake. However, all together, nearly 70 animal species were concerned. About 80% of the reports studied were considered reliable with respect to time and location and were directly observed by identified eyewitnesses. The rest were second hand reports. In 45 cases observers of animal anomalies expected an impending earthquake and notified authorities. The animal precursor was reported before the quake. In the majority of cases the anomalies observed gained importance only after the quake. For example, this happened in the case of a woman from the village of Daodi, in the district of Fengnan. Before she died of her injuries she said that on the evening before the quake the children had insisted that mice were behaving so strangely that an earthquake may be imminent. The woman considered this as nonsense and did not react. Unfortunately the quake arrived and the children did not survive. 

The general abnormal animal behaviour observed prior to the Tangshan earthquake matched that observed with other major earthquakes in China. Two categories could be distinguished. One is a reaction of fear in animals, the other a reaction of melancholy. Horses, cattle, and donkeys were frightened, refused to enter their stable, and did not eat. Also pigs and sheep refused to enter their pens, and if already in them, tried to break out. Dogs barked in an exited way, ran around, sniffled on the ground, and evacuated pups from the houses, carrying them in their mouths. Also cats evacuated their kittens, were restless, made a noise, and woke up their owners. Rabbits refused to eat, made a noise, and attempted to evacuate their pens. Chickens refused to enter their stables, and jumped and flew up to high branches of trees. In the pens they were frightened as if a marten were around. Ducks and geese avoided going into water or fled from it, did not eat, and refused to enter pens. Pigeons avoided their loft, and flew frightened in swarms. Fish circulated in shoals close to the surface, and jumped from the water, as well as from an aquarium. Mice formed swarms either to leave the area or to rest motionless. They were no longer frightened of man. 

Before the Tangshan quake there was also some very unusual and dramatic animal behaviour. In the district of Wuning more than 20 eye witnesses observed over 100 fire-weasels running towards a village centre. Later on 10 of them circulated aimlessly around a walnut tree. Five of these were slain by local people. One occurrence also reported before the earthquake was the sighting of approximately 100 mice, who rested quietly on the ground within an industrial plant in Jixian. From the districts of Qian’an and Ninghe large swarms of dragon flies were reported. One extended for 100 meters. From the apiaries of the district of Qianxi and the people’s commune Miaolingtou nearly all of more than 100 and 30 beehives, respectively, were evacuated by the bees before the earthquake. They all took off in a given direction. Near the harbour town Tingbo a large oil tanker became literally covered by dragon flies, butterflies, grasshoppers, crickets and cicadas. They could be touched by hand.

Around the rice fields of the village of Hangu and also in the district of Qianxi the intensive quaking of many frogs was a regular evening spectacle. However during the night preceding the earthquake no tone was heard. An effort, by a local person, to capture frogs that night failed. The frogs seemed to have interrupted their activity and to have moved away. The day before the quake several hundred snakes were observed in the district of Tianjin rolled up and calm near a water pond. When startled, the normally shy snakes entered first the water, but then immediately returned to the ground assuming the same position. Near the city of Tianjin and near the people’s commune Xiyingmen large swarms of bats were observed during the bright day preceding the earthquake. The breeding station for sable in Dashantou had a stock of 415 animals, when, two hours before the earthquake, the keeper was alarmed by excessive noise. He suspected that an animal had broken out, but he discovered nothing special besides unusually excited animals. 

It was found that the unusual animal behaviour before the Tangshan earthquake was concentrated on the areas where the quake was strongest (more than seven on the Richter scale), and where the critical fault lines were concentrated. When, within the region with more than seven on the Richter scale, only areas within 5 km from the fracture lines are evaluated, an interesting observation can be made. The density of abnormal animal behaviour there is found to be five times higher than in the rest of the affected central area. This observation, the correlation of animal anomalies with fault lines coincides with similar ones from other Chinese earthquakes, for example that of Haicheng.

The abnormal animal behaviour around the Tangshan earthquake started (as Figure 5, right, shows) approximately two days before the earthquake event and reached its highest level eight hours before the quake. For the districts Tangshan, Tianjin and Langfang time dependent graphical distributions were compiled [27] (Figure 5, right). Earlier than two days before the quake, there was only irrelevant activity. An increase of the incidence of abnormal animal behaviour by factors of 68, 66 and 36 respectively was observed towards the quake. This is very significant. With the Haicheng earthquake, in contrast, the abnormal animal behaviour started two months ahead of the quake and clearly intensified 15 days before. The reason may have been an imminent earthquake event, which, for some geophysical cause, was finally delayed.

The above analysis of animal unrest before the Tangshan earthquake shows that the animal phenomenon was relevant and could have been useful for earthquake prediction (together with physical precursors) like in the case of Haicheng. In fact, in one of the counties hit by the Tangshan quake the earthquake alert system actually did work. It was in Quinglong county. This example shows an exception to the apparently politically motivated turbulence during the failed Tangshan earthquake prediction efforts.

## 9. The Successful Earthquake Prediction Strategy of Qinglong County

The UN global program for the integration of public administration and the science of disasters conducted a detailed study of the pre-earthquake strategy of Qinglong county [32,33]. 

Like other counties of the region later affected by the Tangshan earthquake, Qinglong had started a massive education campaign in anticipation of a major earthquake. A total of 70,000 books and 14,000 posters were distributed and 120 slide shows presented. Sixteen county-, 42 commune-, and 442 village-level observation stations were established and staffed. The lay stations watched for changes in water properties, animal behaviour, geo-electricity and geo-magnetism. Two weeks before the Tangshan earthquake the young administrator Wang Chunqing participated in a conference organized by the State Seismological Bureau, during which warning of an imminent earthquake was given. The county acted by relocating school classes and moving teaching and business activities outdoors. An around-the-clock earthquake watch was organised. While the instrument group (electric and magnetic measurements) did not see any unusual changes nocturnal animals like weasels and rats were observed to move in full daylight. Also water springs became muddy. By 26 July, one day before the disaster, many of Qinglong’s 470,000 residents had moved into tents, the rest slept with open doors and windows as a strategy for fast escape. Even though more than 180,000 buildings collapsed, only one person died and that was of a heart attack.

The tragic Tangshan earthquake experience does not disprove abnormal animal behaviour as potential earthquake precursors. In contrast, it adds valuable information.

## 10. Satellite Information on Heat Anomalies and Heat Island Phenomena Preceding Earthquakes

If earthquakes are preceded by precursor phenomena such as animal unrest, some energy is needed to generate them. Since, during energy conversion, energy is finally converted into heat, unusual heat generation should consequently precede major earthquakes.

**Figure 6:**
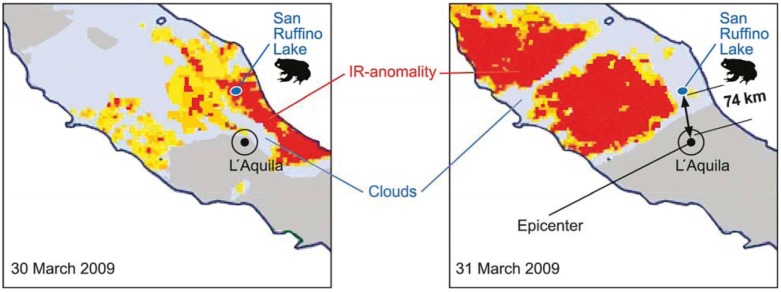
Infrared anomaly seen from a satellite (infrared anomaly distribution adapted from [48]) over central Italy before the Abruzzo earthquake. The left image was taken on 30 March, the right one on 31 March (the quake happened on 6 April, 3.32 A.M.). The arrows in the figures indicate the site of animal (toad) observation and the epicentre respectively (the infrared absorbing clouds are also indicated).

Past earthquakes can be analysed for pre-earthquake thermal anomalies using recorded satellite data (e.g., NOAA-AVHRR thermal infrared time series datasets, passive microwave SSM/I sensor datasets from DMPS satellites). This has been done for a series of more recent earthquakes, such as Bhuj (Gujarat, India, M = 7.7, 26 January 2001), Boumerdes (Algeria, M = 6.8, 21 May 2003), and Bam (Iran, M = 6.6, 26 December 2003) but also for several additional earthquakes, larger than magnitude six on the Richter scale, in China (Xinjang, M = 6.4; 24 February 2003), Afghanistan (Hindukush, M = 6.2, 7.2; 3 March 2002) and Pakistan (Kalat, M = 6.1, 4 March 1990) [34,35,36,37,38,39,40,41,42,43,44,45,46,47]. The astonishing result is that such thermal anomalies actually appear to exist. They show up one week, several days or hours before the earthquakes. The recent Abruzzo earthquake of 6 April 2009, was preceded by infrared anomalies starting 13 hours before the strongest foreshock (30 March) and showing a maximum extension on 31 March, five days before the main earthquake event (it occurred on 6 April at 3.32 A.M.) [48,49,50]. These latter studies are especially relevant, because for the same earthquake reliable animal precursors were observed by Rachel Grant [51]. She was investigating colonies of common toads at San Ruffino lake 74 km north of the epicentre near L’Aquila. Five days before the earthquake the number of toads fell by 96%. While spawn was found at the site up to six days before the quake, no spawn was laid afterwards, until after the quake period. An interruption of the mating activity is an entirely unusual phenomenon. What happened five to six days before the Abruzzo earthquake in terms of the infrared anomaly seen from a satellite? On these days the infrared anomaly actually appeared for the first time in the earthquake region, as seen from Figure 6 (distribution of infrared anomaly adapted from [48]), and spread across central Italy (dark (red) areas in Figure 6 left: 30 March, right: 31 March). The site of the toad studies and the epicentre are indicated. Since clouds (indicated grey areas) obscure the infrared anomaly, the phenomenon is only partially seen. Nevertheless, it appears that the infrared anomaly passed over the site of the toad observation on the critical days of abnormal animal behaviour. There is apparently a correlation.

What is already known about satellite observed infrared anomalies? The observed anomalies suggest a rise of temperature by a few degrees and up to 10 degrees. This is under the hypothesis that the surface did not change its radiation properties (emissivity). The Bhuj earthquake of 26 January 2001 in Gujarat, India, was, for example, based on such an assumption, preceded by a 4 °C or 5–6 °C temperature anomaly, according to different evaluations [25,45] just three days before. On the basis of an analysis of night time maps a thermal anomaly of 5–10 °C was identified two days prior to the 22 May 2003 earthquake of Boumerdes. Four days before the 26 December 2003 earthquake of Bam, and with a peak at two days before the earthquake a 5–7 °C, in some areas a 8–10 °C thermal anomaly was deduced from night and daytime temperature data [45]. The devastating 17 August 1999 earthquake of Izmit in Turkey (magnitude 7.6) was apparently preceded by a 2–6 °C temperature anomaly, which started 10 days before the quake around the epicentre and then spread over western Turkey. Towards the time of the quake the temperature anomaly increased to 6–10 °C. In all observed cases of temperature anomalies, they apparently disappear on the day of the quake. It is for this reason that heating of the ground from below by the observed temperature difference is excluded as a possible phenomenon. The cooling process of the soil would simply take much longer than one day. In contrast, a local greenhouse effect produced by gases escaping from underground prior to the earthquake provides a reasonable explanation [36]. In this case the presence of the gas would change the emissivity of the ground studied for infrared radiation emission and also contribute to heating, via its own heat capacity, and via the greenhouse effect. The temperature measurement would have to be recalibrated. If released gases and aerosols are responsible for higher infrared emission, they would also have to absorb infrared radiation between 8 and 14 μm, where thermal infrared cameras work. In this range stretching vibrations of C-O, C-N, C-C chemical bonds, as well as rocking vibrations of C-H and N-H bonds occur.

The reported temperature anomalies [34,35,36,37,38,39,40,41,42,43,44,45,46,47,48,49,50] are based on the assumption of unchanged emissivity of the earth’s surface concerned. In fact there may be a rise in temperature coinciding with an emission of gases and aerosols from the earth. Temperature measurements themselves have to be put on a much more reliable basis in terms of meteorology. There is, for example, a considerable difference between air temperature (measured in ventilated boxes) and surface temperature (from satellite measurements, which determines the temperature of the surface and surface-near air via radiation). Their relation is not yet convincingly addressed, but, since meteorological temperatures are used for comparison, such information is needed. All together, there seems to be no other reasonable explanation for the infrared anomaly before earthquakes than an escape of gases exhausted from underground with parallel temperature changes. For a further clarification, wavelength dependent infrared measurements will be needed together with more sophisticated temperature measurements. In addition the surveillance of radiation emission should be extended towards the onset of the microwave region, so that clouds can be penetrated better.

What relation do these modern indications on exhalation of gases before earthquakes have with the “pneuma” concept of Greek and Medieval philosophers and to reported animal earthquake precursors from popular tradition?

## 11. Discussion

In 1700 B.C., a severe earthquake levelled a small temple overlooking the area of the Minoian cultural centre of Knossos on the island of Crete. Careful archaeological research revealed, that the persons slain had just performed a human sacrifice, rare for the Minoian civilisation. The priest in charge had obviously tried to avert the natural disaster. Theoretically such a conclusion may be debated, but the rareness of human sacrifices in Minoian culture and other discussed circumstances are conducive to the later Dionysos/Bacchus cult, which indeed concentrated on disaster anticipation. What were the precursors recognised? In order to answer this question two strategies were followed. One was to track down the supposed following up of religious traditions of the Dionysos/Bacchus cult, which centred around the worship and control of natural forces. The second was to understand on what natural phenomena the Greek philosophers were basing their understanding of earthquakes. The conclusion reached is that the priest was judging atmospheric anomalies, related to a gas escaping from underground and to the formation of local heat islands, with obvious consequences for animal behaviour. Modern satellite observations of heat anomalies preceding larger earthquakes appear to identify temperature anomalies in relation to gas emission, which appear to be consistent with the reported ancient observations. 

That the Minoan culture had experience with disaster anticipation is also supported by the complete absence of victims, in the excavated settlements, of the disastrous volcanic eruption of the Santorin volcano around 1650 B.C. However, the kind of precursors observed is not known. Searching for the religious background and heritage of the earthquake averting ritual, we have identified the Bacchus cult in Rome with its ritual human sacrifices. They were aimed at calming down the god who controls natural forces. A painting of Bacchus in front of the Vesuvius (Figure 1) was reinterpreted to show animal unrest before earthquakes and volcanic eruptions, which may underline the significance of animal precursors. Throughout all antiquity natural philosophers have identified themselves with an earthquake concept involving the escape of a warm gas “pneuma” from underground prior to and during the earthquake. Erroneously ancient philosophers thought the “pneuma” was the source of the earthquake. We have seen that it could be interpreted as a precursor and accompanying phenomenon of earthquakes. As such it is also in agreement with phenomena reported from popular tradition from earthquake regions all over the world. Atmospheric phenomena, animal precursors and luminous phenomena, all could be related to gases and electrically charged aerosols escaping from underground [6].

It has been shown that modern satellite observations of infrared anomalies before major earthquakes suggest the same conclusion, that gases escape and generate local greenhouse effects. However they need, of course, to be better evaluated and matched with meteorological evidence. All together the assumption, that the satellite detected thermal anomalies producing escaping greenhouse gases are related to the ancient “pneuma” concept of escaping gases provides a reasonable working hypothesis, which could be tested with appropriate experiments.

It remains now to be tested whether the cross linking between atmospheric changes and animal anomalies is consistent.

Experience up to now with thermal anomalies before earthquakes suggests that they appear in the range of 10 days, one week or a few days or hours before the earthquake, increase towards the disaster and disappear within one day after. Reported animal anomalies cover approximately the same time period. A well known report from the 4th century B.C., for example, cites that five days before the destruction and drowning of the Greek harbour town of Helice, all animals, especially rats, snakes, weasels, millipedes and worms started to leave the city [52]. From 78 earthquakes with animal anomalies evaluated [6], ten percent report animal behaviour ten to three days before the disaster. Periods of one to three days are reported by 24 percent of documents. On the average, worldwide, animal anomalies are observed 21 hours before the earthquake disaster. Remarkably the time period in Europe (18.5 hours), China (25.5 hours), Japan (23.5 hours) and the rest of the world (21.7 hours) are very similar [6]. It can therefore be said, that thermal anomalies and abnormal animal behaviour occur within a similar time period before a major earthquake. The experience with animal anomalies preceding the Tangshan earthquake also appears to support this conclusion (Figure 5, right). They occurred within two days before the quake. There are numerous reports from all over the world saying that earthquakes are preceded by unusual heat and a very special dry atmospheric condition (earthquake weather) [6]. This seems now to be directly supported by the satellite observations of significant temperature anomalies preceding earthquakes. A temperature increase of several degrees is definitively felt by people and a greenhouse climate laden with gases and aerosols may be experienced as additional burden. Popular earthquake tradition also communicates that an earthquake may drastically change the weather [6], for example from foggy and rainy before to clear afterwards or the other way around. This tradition is, for example communicated from Chile and Peru. This situation may simply reflect the fact that the thermal anomaly observed from satellites disappears within the day of the earthquake. With the temperature anomaly also the greenhouse effect and its gases and aerosols are expected to disappear. This may indeed also drastically change the weather. 

In order to find out whether temperature anomalies and animal anomalies coincide it was attempted to select an earthquake, for which thermal anomalies were reported and to look for coinciding animal anomalies, which have been reliably documented. We selected the Abruzzo earthquake of 2009 for which infrared anomalies (Figure 6) are documented and during which professional animal (toad) observations were performed [51]. When the infrared anomaly touched the animal observation area six to five days before the earthquake the toads actually ceased their mating behaviour. As an additional example we may select the Bhuj earthquake in Gujarat, India. During the period when it occurred in 2001 there were no clouds in the entire earthquake region so that thermal anomalies could be reliably documented and studied (e.g., [25,36]). We looked for reports on coinciding animal precursors. Today, such a search is possible via the Internet. It was found that the Bombay Natural History Society, on behalf of the Union Ministry for Environment and Forestry, had conducted a survey on earthquake related animal behaviour for this earthquake [53]. The information obtained from amateur observers turned out to be of mixed nature. Again and again there were reports of excited dogs and birds. In Teras, 80 km west of Bhuj, the epicenter, it was observed that during the night preceding the earthquake, which hit in the morning, the peacocks were hysterically crying. Dogs were also barking and donkeys crying before the aftershocks later during the day. However, not all reports on animal behaviour were positive. Some domestic animals simply did not react at all until the earthquake hit. Our tentative explanation is that many of the domestic animals today live in an environment polluted with noise, smog and odours from traffic, people and technical installations. They are flooded with signals and stimuli. Maybe some of the animals were not able to distinguish any more between an artificial source of pollution and the atmospheric changes accompanying an approaching earthquake. We therefore looked for an area in the region affected by the pre-seismic heat anomaly, as seen from the satellite, but which had remained essentially spared from the influence of modern civilization. There is a national forest in the area which was touched by the satellite monitored infrared anomaly, preceding the earthquake, and which was subsequently hit by the earthquake. It is the famous Gir forest, which harbours the last Asiatic lions in an entirely natural environment. On the Web page on the Asiatic lion [54] there was direct information about the events preceding the large Bhuj earthquake. The vice conservator of the Gir forest, Maresh Singh, and a few members of the staff of this 1,382 square kilometre large park became eye witnesses of the following phenomena: During the night from the 25 to the morning of the 26 January 2001, when the earthquake occurred, they observed that animals were involved in unusual movements. The tranquillity of the Gir forest was shaken by the roaring and cries of animals. Singh said that the lions, leopards, gitas and deer of the jungle cried loudly and ran around erratically. The roaring of lions was deafening and they ran around with their tails highly erect. The staff which were working in the area could initially not understand this unusual behaviour. Singh camped that night also outside in Jamwa and observed how snakes left their holes and attempted to move up into the trees. 

Since the documented earthquake related temperature anomaly, apparently caused by a greenhouse effect generated by gases escaping from underground, affected this Indian national forest, there is obviously a direct connection with the anomalous animal behaviour. Animals recognise such signals from the underground, which indicate a clearly changing environment, which is frightening them. Behavioural scientists know that the erect tails of cats signal aggression and threat. With the erection of the tail they apparently attempt to increase their size. In an earlier study [6] we gave evidence that it may be positively charged aerosol particles which are responsible for the aggression of certain animals before earthquakes (e.g., Figure 2), since they generate the Serotonin irritation syndrome (also known to produce aggression and restlessness in connection with certain dry winds). The behaviour of lions from the Gir forest fits into this picture. 

The early Indian scientist Varahamihira (505–587 A.D.), by the way, already knew about abnormal animal behaviour and interestingly also about a strange weather anomaly preceding an earthquake in his homeland. In his work Brihat Samhita (The Great Compilation) he speaks of unusual cloud formation a week before its occurrence. It may be the same phenomenon recognized now as infrared anomaly from satellites.

Most earthquake researchers and geologists today are still very reluctant to accept phenomena from the popular tradition as credible. The consequence is that they remain almost unexplored. One reason for the reluctance is that they understand the earthquake as a movement of plates, and cannot understand why there should be a significant energy release before. We have dealt with this question in another study [55] and shown that this is not a contradiction. Within irreversible thermodynamics a highly energy loaded system can release energy in the form of non-linear phenomena (e.g., chaotic, multi-step-, oscillating, pulsing processes, pattern formation) before approaching equilibrium (the actual plate moving earthquake event). Such behaviour can be calculated within the frame of non-linear irreversible thermodynamics and means that, during the initial phase of a large earthquake, still existing feedback processes produce some local order and some distributed disorder (entropy), which dissipates part of the huge energy reserve stored and subsequently released in the large earthquake. Spatially distributed pressure changes may arise underground. They can lead to a series of pressure dependent physical-chemical phenomena, e.g., changes in chemical equilibrium, which may release gases and electrical charges from boundary layers of interfaces. The liberated species may penetrate to the earth’s surface and eventually cause animal anomalies, because they can change the composition of the atmosphere above ground. After this initial phase, after and during the release of the classical “pneuma” gas, the earthquake event may approach equilibrium by allowing the dramatic sliding movements of the tectonic plates. The involvement of electrical charges and electrical fields is obvious from many reports on earthquake lights. They were also observed in Tangshan and caused a conductor to stop a train during night time ahead of the quake event. Hours before this quake event technically trained people also observed that switched off neon lights in industrial installations sporadically emitted light. Electrical charges can also explain why fish, water fowl, frogs and toads have problems with their humid environment: they induce electrical currents which are sensibly felt and enable electrochemical reactions, which may change the chemical composition of water. 

Our hypothetical conclusion is that priests and early scientists from Greek antiquity were attentive observers of weather phenomena and had a clear concept of the atmosphere under normal conditions (which was at that time still unpolluted by industrial aerosol particles and free of condensation trail forming airplanes, which now affect the atmosphere worldwide). Occasionally, and under favourable geological conditions they saw smog rising from the underground, which cumulated in earthquake events. They based the “pneuma” theory of escaping gases on such observations and had thus also an explanation for unusual animal behaviour. They attempted to avert earthquakes through sacrifices, since they considered them a punishment from the gods. Therefore observation of earthquake precursors became important and was the task of priests.

Modern seismology is essentially based on sensitive mechanical instrumentation and the expectation that any mechanical soil movement during the quake can be monitored from a considerable distance. The accepted mechanism for earthquake generation gives little room for localized gas emission or nervously behaving animals. Satellite based infrared emission anomalies may eventually re-confirm the “pneuma”-theory of ancient natural philosophers, with the restriction, of course, that the “pneuma”-gas is not causing the earthquake but simply preceding and accompanying it. With such a concept, unusually behaving animals before earthquakes may become an understandable phenomenon, provided substantial experiments are performed, along the line discussed in the following section. 

## 12. How to Proceed in the Future

It is hoped that our analysis on attempted earthquake forecasting during a period of more than three millennia, throughout antiquity, as well as in more modern times during the Chinese earthquake prediction initiative, will motivate some scientists to look closer into the relationship between thermal anomalies, animal behaviour and earthquakes. A first step, besides intensifying and refining satellite observations, could be to follow the idea of ancient scientists such as Aristotle, Poseidonios or Theophrastus and to include earthquake studies in the field of metrological science. Nowadays an immense number of meteorological data from a wide area is daily evaluated by computer programs and used to predict the weather in specific locations. This procedure is especially requested for the prediction of unusual weather conditions like storms. Earthquakes appear, at least in certain cases, also to generate unusual weather conditions. When predicting temperature and weather data for specific areas known for earthquakes, meteorologists should subsequently and systematically check whether their prediction was correct and what their error was. They should specifically look into temperature and weather anomalies, which they cannot explain on the basis of meteorological experience. Most work could be done on an automatic basis using appropriate computer programs analysing the vast quantity of meteorological data which is continuously generated. This way, and including improved satellite data whenever possible (during clear weather), temperature and weather anomalies coinciding with earthquakes could be more accurately identified. If they were to be recognized in time before the earthquake, experts could be informed and activated to start immediately observing and monitoring animals in zoos, on farms and in the wild. Suitable animal observation strategies with high statistical value should be prepared (e.g., variations in egg production, sound in bee hives, permanence of animals in pens, swarm formation in wild species *etc*.) A narrow time window of one to several days for intensive animal and geophysical studies would become available which gradually could lead to accumulation of valuable information. Funding, for such initiatives, should be prepared in advance. Biologists may then find out how animals react and what they sense and physicists may support them by analysing gas and aerosol composition in such developing earthquake epicentres. New knowledge will become available on earthquake phenomena and we may also understand more about antique earthquake concepts. This may, hopefully, over the years increase our understanding of earthquake precursors to a standard sufficient for averting danger for people and critical industrial installations. In any case we will then finally know whether the two (of four) earthquake precursors, animal and fog anomalies, which Pliny the Elder described nearly two thousand years ago, and which modern science today still ignores, are based on actual natural observations.
